# Prevention of mental disorders: evidence, challenges and opportunities

**DOI:** 10.1186/1741-7015-12-75

**Published:** 2014-05-09

**Authors:** Felice N Jacka, Nicola J Reavley

**Affiliations:** 1 IMPACT Strategic Research Centre, Deakin University, Geelong, Australia; 2 Department of Psychiatry, University of Melbourne, Melbourne, Australia; 3 Melbourne School of Population and Global Health, The University of Melbourne, Parkville, Australia

**Keywords:** Prevention, depression, mental disorders, mental health, policy

## Abstract

Modelling studies suggest that less than 30% of the burden of mental disorders can be averted, even with optimal care and access to services. This points to the need to reduce the incidence of mental disorders, utilising evidence-based prevention strategies and policy action. In this cross-journal article collection (http://www.biomedcentral.com/series/PMD), the case for prevention is made by identifying initiatives with established efficacy, as well as opportunities and targets for the prevention of mental disorders in early life, in the workplace and at the population level. These articles provide reviews, systematic and narrative, outlining the evidence base for prevention approaches, as well as comment and debate designed to prompt discussion and a reconsideration of strategies for prevention. Barriers to expanding the research into prevention include the reluctance of governments and funding bodies to invest in research and policy action that may take many years to manifest benefits. The case for the cost-effectiveness of preventing mental disorders needs to be strongly argued and new cross-disciplinary, intersectoral initiatives and policies developed for the prevention of mental disorders across the lifespan.

## Editorial

In 2004, Andrews and colleagues estimated that even with optimal care and service delivery, less than 30% of the burden of disease attributable to mental disorders could be averted [1], making a strong case for an increase in access to effective treatments to be paralleled by a focus on reducing the incidence of mental disorders. Years later, despite the efforts devoted to increasing access to services, the burden of mental disorders at the population level, at least in Australia, appears unchanged [2]. At the same time, research funding focused on the prevention of mental disorders, which is consistently rated as of the highest priority by stakeholders, remains low and is declining [3]. This is despite evidence for the effectiveness [4] and cost-effectiveness of many prevention programs, including interventions targeting the prevention of suicide, adult and childhood depression, and childhood anxiety [5]. Nevertheless, in recent years there have been increased research efforts focused on reducing the incidence of mental disorders and there is now substantial cause for optimism regarding the opportunities for prevention. Opportunities for prevention exist across the lifespan and in multiple settings, including early childcare and schools, the workplace and aged care settings, as well as at the population level through policy action [6,7].

The first few years of life play a critical role in determining mental health and wellbeing in later life and there is substantial evidence for the effectiveness [8,9] and cost-effectiveness of interventions targeting parenting and childhood [10]. Preventing mental health problems early in life can avert prospective costs associated with crime, lack of education, unemployment and unhealthy and risky behaviours. In this cross-journal article collection focused on prevention (published in *BMC Medicine* and *BMC Psychiatry*), Lewis and colleagues review the extensive literature on the early life programming of mental disorders and offer insights into targets for preventive interventions [11]. The authors stress the importance of addressing risk factors in pregnancy, including poor maternal mental health, unhealthy lifestyle behaviours (diet, related obesity and smoking) and potential teratogenic and neurotoxic exposures, in order to improve child neurodevelopmental, emotional and behavioral outcomes.

Similarly, with 60% of adults in the workforce, and good evidence for the cost-effectiveness of many workplace-based interventions [10], the argument for addressing mental health problems and improving wellbeing in the workplace is a strong one. Tan et al. [12] offer the first systematic review and meta-analysis of universal preventive interventions in workplace settings. They conclude that universally delivered workplace mental health interventions, particularly those based on Cognitive Behavioral Therapy, can reduce the level of depressive symptoms among workers.

This is followed by a debate arguing for the integration of psychological, medical and public health approaches to good mental health at work [13]. Such an integrated approach would protect mental health by reducing work–related risk factors, promote mental health by developing the positive aspects of work as well as worker strengths and positive capacities, and address mental health problems among working people regardless of cause. In their debate, LaMontagne *et al*. also emphasize the need to move away from the current emphasis on interventions directed towards individuals to those that also encompass organizational changes, such as those focused on job stress prevention.

Finally, Jacka et al. provide an argument supporting the need to integrate physical and mental health promotion and prevention initiatives, with a focus on improving the food environment through policy action [14]. This commentary arises in response to the rapidly growing, consistent and compelling evidence base suggesting that unhealthy diets are risk factors for common mental disorders and dementia, whilst healthy diets are protective. Such an understanding, whilst in its nascent stages, reinforces the imperative for governments to take urgent action to address ‘obesogenic’ environments in western and developing nations, arising largely as a result of the globalization and activities of the food industry.

### Challenges and opportunities

Governments and research funding bodies, somewhat understandably, give preference to short-term interventions that can demonstrate effects and generate reports within a three-year time frame, whereas prevention interventions ideally require long-term follow up periods. Certainly prevention initiatives in health are less appealing to governments due to the fact that costs, whether political or economic, are incurred upfront, whilst benefits likely manifest years after a current government may have changed hands. Moreover, in the case of mental health, the determinants of poor mental health largely exist outside of the health sector making effective initiatives more complex and challenging. This highlights the imperative for intersectoral engagement and cross-disciplinary action, as well as comprehensive governmental policies to address the prevention of mental disorders at a population-level.

In order to continue to progress the prevention and mental health promotion agenda, there is a need for governments, other policy makers and business leaders to fully recognize the impact of poverty and social disadvantage, environmental determinants of health and educational and workplace policies on the mental health of the population. In order for this to happen a strong economic argument can be most persuasive. In some jurisdictions the message is being heard; in the UK, the health benefits and economic savings of evidence-based interventions to prevent and intervene early with mental illness and promote good mental health are highlighted in the government’s 2011 strategy paper, *No Health Without Mental Health*[15]. Similarly, the new Early Intervention Foundation in the UK, launched with cross-party support in 2013, targets early life risk factors and aims to support social and emotional learning in high-risk children and families in order to improve long-term mental, behavioral, social and economic outcomes. This foundation arose in recognition of the strong economic case for investing in early life and the need for long-term political and financial commitment to programs whose benefits will accrue over extended periods. Such integrated approaches to promoting mental health are welcome, but are also required in other age groups and settings. Certainly the early years are not the only opportunity for the prevention of mental disorders, as these *BMC Medicine* and *BMC Psychiatry* papers demonstrate.

This prevention-themed cross-journal article collection arose as an initiative of the new Alliance for the Prevention of Mental Disorders (APMD), which was launched in Australia in 2013. The aim of the APMD is ‘to support a population health approach to the prevention of mental disorders and promotion of emotional wellbeing’ through advocacy, support for research, collaboration, capacity building and knowledge translation, and to provide a single source of authoritative advice to policy makers. A primary aim of the APMD is to work towards a mental health research and services system that has a central focus on prevention as well as on access to treatment, recognizing that prevention and promotion interventions may be the most effective and cost-effective approaches to the considerable global burden of disease imposed by mental disorders. Further information can be found at http://www.APMD.org.au.

The articles in this prevention-themed cross-journal article collection outline the evidence base for prevention interventions across the lifespan and in a range of settings, adding further weight to the compelling case for prevention to be an important focus of policy and research actions. They provide guidance for policymakers and other stakeholders wishing to implement prevention interventions that already have a strong evidence base. They also aim to inform future research directions by pointing to many further opportunities for developing and implementing interventions designed to reduce the considerable burden of disease related to mental disorders.

## Competing interests

The authors declare that they have no competing interests.

## Authors’ information

FNJ is President and NJR Vice President of the *Alliance for the Prevention of Mental Disorders (APMD)*. Both are Australian mental health researchers with a strong interest in prevention.

## Note

All articles in this article collection have been independently prepared by the authors and have been subject to the standard peer-review processes of the journals.
